# Crosstalk between the Gut and Brain in Ischemic Stroke: Mechanistic Insights and Therapeutic Options

**DOI:** 10.1155/2022/6508046

**Published:** 2022-10-11

**Authors:** Wenjing Huang, Luwen Zhu, Wenjing Song, Mei Zhang, Lili Teng, Minmin Wu

**Affiliations:** ^1^Heilongjiang Administration of Traditional Chinese Medicine, Harbin 150000, China; ^2^The Second Affiliated Hospital of Heilongjiang University of Chinese Medicine, Harbin 150000, China; ^3^Heilongjiang University of Chinese Medicine, Harbin 150000, China

## Abstract

There has been a significant amount of interest in the past two decades in the study of the evolution of the gut microbiota, its internal and external impacts on the gut, and risk factors for cerebrovascular disorders such as cerebral ischemic stroke. The network of bidirectional communication between gut microorganisms and their host is known as the microbiota-gut-brain axis (MGBA). There is mounting evidence that maintaining gut microbiota homeostasis can frequently enhance the effectiveness of ischemic stroke treatment by modulating immune, metabolic, and inflammatory responses through MGBA. To effectively monitor and cure ischemic stroke, restoring a healthy microbial ecology in the gut may be a critical therapeutic focus. This review highlights mechanistic insights on the MGBA in disease pathophysiology. This review summarizes the role of MGBA signaling in the development of stroke risk factors such as aging, hypertension, obesity, diabetes, and atherosclerosis, as well as changes in the microbiota in experimental or clinical populations. In addition, this review also examines dietary changes, the administration of probiotics and prebiotics, and fecal microbiota transplantation as treatment options for ischemic stroke as potential health benefits. It will become more apparent how the MGBA affects human health and disease with continuing advancements in this emerging field of biomedical sciences.

## 1. Introduction

Stroke is the second leading cause of mortality and a significant contributor to disability globally [1]. Strokes come in two different varieties: ischemic and hemorrhagic. Ischemic stroke (IS) is caused by a thrombus or embolus blocking a cerebral artery, whereas hemorrhagic stroke is caused by a ruptured cerebral vessel [2]. The most prevalent type of stroke worldwide is IS, with 24.9 million cases annually, which imposes a considerable burden on society [1]. Due to its complicated pathogenesis, it exhibits refractory properties, particularly regarding the secondary damage caused by an early ischemia time window and reperfusion [3]. Therefore, the development of measures to lower the prevalence of IS and its detrimental consequences is highly warranted. Recent research has demonstrated that the gut microbiota regulates the pathogenesis of IS via the microbiota-gut-brain axis (MGBA) [4, 5].

The gut microbiota and gut microbiome refer to the collection of all the gastrointestinal (GI) microorganisms and their genetic material, respectively. These commensal microorganisms include eukaryotes (fungi and parasitic helminths), prokaryotes (bacteria and archaea), and viruses [6]. The community of microbes that resides in the GI tract is the largest and most diverse of all the communities of microorganisms and has received a great deal of attention [6]. Naturally, there is mounting evidence for a two-way exchange of information between the central nervous system (CNS) and the GI microbiota, also known as the MGBA, which has been demonstrated to be a significant contributor to the physiology of the brain. The gut microbiota can affect the cardio-cerebral-vascular system, immune system, gut function, and physiological activities through signaling molecules and bioactive metabolites. Additionally, the prevalence of IS are closely related to unchangeable factors (sex, age, and genetic predisposition or pathological alterations) and modifiable factors (hypertension, diet, lifestyle, obesity, hyperlipidemia, smoking, and abnormal blood glucose) [7, 8]. They all significantly impact the diversity and abundance of the gut microbiota.

Here, we concentrate on the gut microbiota's current role in the pathogenesis of IS and how it affects its associated risk factors. We also discuss the potential of the gut microbiota as a novel therapeutic option for the prevention and treatment of IS.

## 2. Healthy Microbiome and Dysbiosis

The respiratory tract, skin surface, genitourinary systems, and GI tract all include commensal microorganisms. The gut contains commensal microbes, which comprise approximately 95% of the human microbiome. There are over 100 trillion bacteria, representing up to 5000 different species, and they weigh approximately 2 kg in the human gut, which contains ten times more microbial cells than the entire body [9]. More than 100 bacterial phyla constitute the human GI microbiota, with the majority of these species belonging to two phyla, namely, *Firmicutes* (*Ruminococcus*, *Clostridium*) and *Bacteroidetes* (*Prevotella*, *Porphyromonas*), with relatively small amounts of *Actinobacteria* (*Bifidobacterium*), *Proteobacteria*, and *Verrucomicrobia* [10]. It has been established that the ratio of the bacterial species of *Bacteroidetes* to *Firmicutes* significantly impacts health and disease [11]. In addition, it is crucial to emphasize that the gut microbiota is heterogeneous, with microbial density and diversity rising along the GI tract following immunological, chemical, and nutritional gradients [12]. As a result, each species is exceptionally well adapted to carry out particular functions in a specific digestive tract environment [12]. These microorganisms have developed a close, mutually advantageous symbiotic relationship with their hosts during the eons of coevolution rather than passively colonizing their hosts' guts [12]. The host supplies shelter and nutrition for its microbial subtenants, and the microorganisms provide numerous health benefits in exchange [13]. Specifically, the digestion of food, production of metabolites, facilitation of nutrient absorption, and metabolism of xenobiotics and drugs are all positive functions of the intestinal microbiota in a healthy condition that supports host nutrition metabolism [14]. A well-balanced intestinal microbiota maintains a normal intestinal epithelial barrier by maintaining the structural integrity of tight-junction proteins, upregulating mucin genes, and limiting the adhesion of epithelial cells to pathogenic bacteria [15]. It also helps immunological initiation, modulation, and pathogen resistance [16].

Gut dysbiosis, also known as gut microbial dysbiosis, refers to pathological abnormalities in the composition, diversity, and abundance of intestinal flora that affect intestinal metabolism, the immune state, systemic inflammation, and other responses [17]. It is characterized by decreased microbial diversity, fewer beneficial bacteria, or a higher concentration of pathogenic microorganisms. The disruption of MGBA signaling caused by dysbiosis of the intestinal flora usually contributes to alterations in the intestinal structure and increased permeability of the mucosal epithelial barrier, resulting in pathophysiological effects [18]. Specifically, multiple factors inducing gut microbial dysbiosis can lead to leakiness of the intestinal wall, resulting in easier entry of endotoxins, microbial elements, and microbial metabolites into the systemic circulation, ultimately triggering an immune response and exacerbating systemic inflammation [19]. Gut dysbiosis causes T cells to polarize into proinflammatory Th17 (IL-17), Th1 (IFN-*γ*), or *γδ*cells. These cells then migrate from the small intestine to the ischemic brain, where they cause infarct damage [20, 21]. Therefore, IS may develop when there is an imbalance in the bacterial species.

## 3. Alterations in the Gut Microbiota during Ischemic Stroke

Microbiome-associated molecular patterns (MAMPs) and metabolites secreted by the microbiome can interact with the mucosal epithelium and intestinal immune cells, stimulate the vagus nerve (VN), or enter the systemic circulation to communicate with the brain and potentially modify neuronal and immune responses [22]. In turn, the parasympathetic and sympathetic nerve fibers of the gut wall convey signals to the brain to influence immune cell and gut motility activity and alter the composition of the gut [23] (this is described in detail below). The commensal microbiota change such that opportunistic pathogens become dominant after IS. This change is most likely caused by the release of cytokines and chemokines produced in the brain, altered intestinal motility and permeability, and mucus production, all of which contribute to dysbiosis. The result of an IS is subsequently worsened by dysbiosis. Acute IS risk factors such as age, hypertension, diabetes, obesity, and vascular dysfunction have also been linked to gut flora dysbiosis [24].

Experimental and clinical research has demonstrated that the gut microbiota composition significantly influences the incidence and outcome of IS [20, 21]. Furthermore, the gut's microbial composition substantially changes during the acute stage of IS [25]. Singh and colleagues showed that cerebral IS results in microbiota dysbiosis, with decreased bacterial diversity in mouse feces including a reduced abundance of *Firmicutes* and an excessive increase in *Bacteroidetes*. These changes are connected to decreased intestinal motility and increased intestinal wall permeability. Additionally, they discovered that microbiota transplantation might improve IS outcomes and impact immunity [21]. Yin and colleagues studied the gut microbes of patients with IS. They found that transient ischemic episodes were primarily associated with opportunistic pathogenic bacteria such as *Enterobacter*, *Desulfovibrio*, and *Megasphaera*, while beneficial bacteria such as *Faecalibacterium* and *Prevotella* were depleted [26]. Another study found that individuals with IS had higher levels of *Atopobium* and *Lactobacillus ruminans*, but *Lactobacillus* levels were decreased [27]. Chen and colleagues found an increased relative abundance of *Bacteroidetes* and decreased relative levels of *Faecalibacterium*, *Oscillospira*, *Lactobacillus*, and *Streptococcus* in a study of monkeys with focal cerebral ischemia, suggesting a correlation with the poststroke inflammatory response [28]. Additionally, they discovered a decline in plasma butyrate concentrations, which may be connected with a reduction in *Oscillospira* and *Faecalibacterium* levels. Monkeys with cerebral ischemia for 6-12 months had decreased plasma levels of short-chain fatty acids (SCFAs), suggesting that persistent gut flora dysbiosis may also influence the generation of SCFAs [28]. Li and colleagues' findings imply that the intestinal flora is related to stroke severity. *Lachnospiraceae*, *Pyramidobacter*, and *Enterobacter* were increased in patients with mild stroke, but *Ruminococcaceae* and *Christensenaceae* were increased in patients with severe stroke [29]. Therefore, dysbiosis not only develops after stroke but also plays a role in its onset.

Benakis et al. [20] reported the relationship between the gut microbiome and the neuroinflammatory response to IS for the first time. They found that the symbiotic gut microbiota protects the brain by controlling immune cells in the small intestine; bacterial priming of dendritic cells leads to the growth of Treg cells, which suppress IL-17+*γδ* T cells. Additionally, they showed that T cells move from the intestines to the meninges, where they control the neuroinflammatory reaction [20]. Studies have indicated that lipopolysaccharides (LPS) may play a key role in chronic systemic inflammation after stroke [30], and elevated levels of plasma LPS or inflammatory cytokines are strongly connected with the overgrowth of *Bacteroidetes* [28]. The higher levels of proinflammatory tumor necrosis factor (TNF-*α*), interleukin-6 (IL-6), and interferon-gamma (IFN-*γ*) in the plasma of focal cerebral ischemia monkeys suggest both intestinal microecological dysregulation and chronic systemic inflammation following cerebral infarction [28]. As a result, chronic systemic inflammation and the poststroke gut microbiota may be potential stroke therapeutic targets.

## 4. Mechanism of the Interactions between the Gut Microbiota and Brain

**Figure 1:**
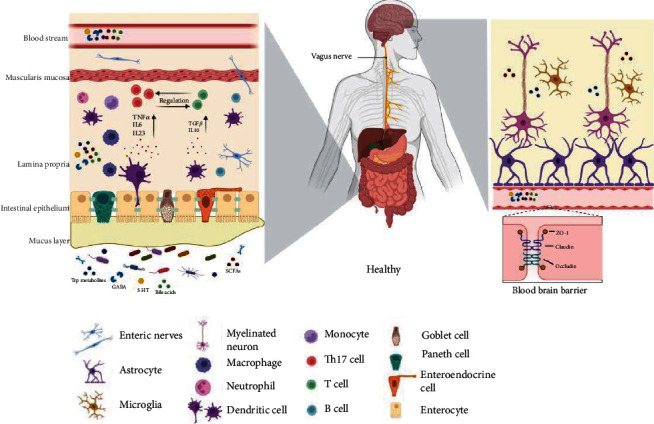
An illustration of the MGBA in the healthy individual. In ENS, the gut microbiota affects how the brain works by releasing various signal molecules that may go to target areas through systemic circulation. Through the ANS, the CNS physiologically controls the GI tract; in turn, the gut provides feedback to the brain to establish a bidirectional link.

The CNS, neuroendocrine, immunological, and autonomic nervous system (ANS) are all involved in the MGBA, which links the brain and the gut via direct and indirect channels. What is known is that constant communication within the MGBA maintains particular aspects of homeostasis, partly via signals originating from gut microbes. This exchange is bidirectional; hence, the gut microbiota can influence the host by altering homeostasis components in both directions. The ability of bacteria to produce neuroactive molecules that promote the communication of the MGBA is becoming clearer (Figure 1).

### 4.1. Neural Pathways

The bidirectional connection of the MGBA occurs along two distinct neuroanatomical pathways. One is the direct gut-brain communication that occurs through the spinal cord's VN and ANS. The other is communication between the spinal cord's VN and ANS and the gut's enteric nervous system (ENS) [31]. The VN is a mixed nerve that connects to the brain and gut and has anti-inflammatory capabilities through its afferent and efferent fibers. VN afferent fibers can be stimulated by microbiota components either directly or indirectly via ENS. Through the inflammatory reflex, VN afferent fibers can activate efferent fibers. The vagal efferents in the medullary dorsal motor nucleus of the vagus, which are part of the vago-vagal anti-inflammatory reflex loop, have the ability to regulate the levels of proinflammatory cytokines in the circulation [32]. The VN transports approximately 70% of the parasympathetic fibers and innervates the whole GI system as well as thoracic and abdominal organs [33]. In stroke, the *α*7 nicotinic acetylcholine receptor (*α*7-nAchR) plays a vital role as a modulator of cholinergic anti-inflammatory pathways. It has been discovered that endotoxemic mice lacking *α*7-nAchR have higher amounts of TNF-*α*, IL-1*β*, and IL-6 in their systems [34]. VN stimulation activates *α*7-nAchR, which lowers pyroptosis, a type of programmed cell death, and increases blood–brain barrier (BBB) tight-junction proteins such as occluding junctions [32, 35].

The ANS is composed of sympathetic and parasympathetic branches. The parasympathetic nervous system controls mood, memory, and appetite and senses microbial metabolites [36]. GI transit, secretion, and motility are all affected by altered sympathetic neurophysiology, principally due to changes in cholinergic transmission and the contractions of smooth muscle. The sympathetic and parasympathetic systems can impact the neuronal circuitry of the ENS, leading to altered motility, which can affect the delivery rate of probiotics and other essential microbial nutrition to the small intestine and colon [37]. The ENS is made up of the submucosal plexus, which controls water and electrolyte flow, and the intermuscular plexus, which controls peristalsis [38]. Acetylcholine and 5-hydroxytryptamine (5-HT) are two of the ENS neurotransmitters that enteroendocrine cells (EECs) can release in response to elevated microbiota metabolites (e.g., SCFAs). The communication between the microbiota and the ENS can transform luminal metabolites into neurochemical signals that control intestinal physiology and may be involved poststroke. The administration of the *L. rhamnosus* strain JB-1 affected mouse behavior via the vagus nerve and caused changes in the expression of *γ*-aminobutyric acid (GABA) receptors in specific brain regions [39]. Mechanisms for the effects of the *L. rhamnosus* strain JB-1 on ENS function further demonstrate that intact epithelium is necessary to mediate the action of JB-1-derived microvesicles, which in turn reflects the effects of the strain on the ENS. This finding suggests that epithelial components may be paramount in mediating microbial signaling from the intestinal lumen to the ENS [40]. These findings suggest that the gut microbiota mediates the neuronal pathways of the MGBA.

### 4.2. Immune Pathways

The immune system and the CNS are intricately organized systems that govern and manage various bodily activities. They both have similar traits in their operational modes and developmental processes. Our intestinal single-cell layer plays an important role in restricting the contact of the gut microbiome with visceral tissue. To do this, the goblet cells of the epithelium secrete a protective viscous mucus layer. Most host-microbe interactions occur at this lumen-mucosal interface, where molecular interactions between mucus layers and epithelial cells facilitate communication between the gut and immune system by distinguishing between self and nonself antigens, enabling the immune system to recognize potentially harmful pathogens [41].

The activity of cells from the myeloid lineage, including neutrophils, macrophages, microglia, mast cells, natural killer T cells, and lymphocytes (T cells), is called innate immunity and is thought to be the body's main line of defense against potentially infectious organisms [42]. It was determined that the maturation of microglia and maintenance of their healthy functional condition require a varied GI microbiome [43]. In contrast, the lack of diverse host microbiota (i.e., GF mice) in the increased microglia population resulted in defects in microglia maturation and differentiation, changed microglia morphology, and impaired immune responses to bacterial infections [44]. Another study revealed that GF mice exhibit impaired microglial cell maturation and that SCFA production by the gut microbiota may affect microglial cell maturation [45]. Cerebral ischemia-induced neuronal cell death releases damage-associated molecular patterns (DAMPs) [46]. The production of proinflammatory cytokines such as IFN-*γ*, IL-6, IL-1, and TNF-*α* increases as a result of DAMPs and neurotoxin-mediated activation of M1 microglia, which is linked to secondary neuronal injury caused by BBB breakdown [47]. Activation of M2 macrophage subtypes promotes the release of chemokines like CCL13 and CCL14 or cytokines like TGF-*β* and IL-10, which improve the outcome poststroke by enhancing BBB integrity, angiogenesis, and tissue healing [48]. Gut microbiota dysbiosis has been linked to increased plasma levels of proinflammatory cytokines caused by loss of BBB integrity [49]. Therefore, a critical therapeutic target for treating ischemic brain injury could be regulating innate immune responses with the aid of the microbiota and its metabolites.

T and B lymphocytes play a pivotal role in mediating the activation of the adaptive immune response. Immune homeostasis is vitally maintained by regulatory T cells (Tregs). Approximately 10% of peripheral CD4+ T cells are CD25+Foxp3+ Treg cells, and they play a crucial role in the induction of immunosuppression by producing cytokines such as IL-10, TGF-*β*, and IL-35 [50]. IL-10 has anti-inflammatory properties that help to maintain gut homeostasis. According to studies, GF mice have few T helper 1 (Th1) and Th17 cells and IL-22 and IL-17, which causes a reduction in lamina propria- (LP-) associated CD4+ lymphocytes [51]. The ability of dendritic cells to endocytose polysaccharides may help naive T cells expand and differentiate into Th17 and Treg cell subsets. Despite their divergent functional characteristics, the degree of TGF-*β* expression determines the development of naive T cells into Th17 cells and Treg cells. Naive T cells are transformed into Th17 cells by low levels of TGF-*β*, IL-23, or IL-6, whereas high levels of TGF-*β* produce Treg cells [52]. In mice with a proinflammatory microbiota, the poststroke polarization of Tregs by naive T cells is inhibited, and the polarization of proinflammatory IL-17+ *γδ* T cells is promoted [20]. More IL-17 secretions could be released by the accumulating T cells, thereby aggravating brain damage [53]. Singh et al. [21] transplanted feces from mice with IS into germ-free mice and discovered that both Th1 and Th17 cells produced significantly more IFN-*γ* or IL-17 after brain damage in mice. Microbial antigens affect B cell activation and differentiation via TLRs [54]. The significance of regulatory T and B lymphocytes in the neuroprotective pathway following the development of IS requires more research.

**Figure 2:**
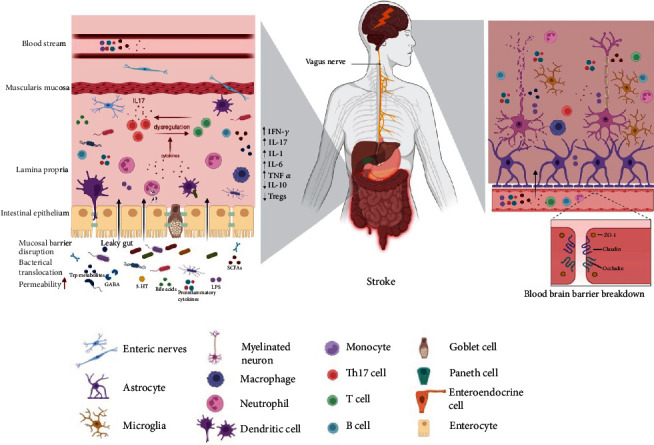
An inflammatory mechanism of the MGBA in IS. By controlling the integrity of the intestinal mucosal barrier, which includes inflammatory cell activation and the production of proinflammatory substances, gut microbiota and their metabolites play a role in the development of intestinal inflammation. Through the expression of TLR, microbial metabolites like TMAO and LPS can also contribute to neuroinflammation and intestinal inflammation. The vagus nerve and blood circulation establish a pathway for intestinal inflammation through which the neurotoxins produced in the gut can reach the brain. The specific manifestations of microbiota-mediated neuroinflammation include the breakdown of the blood-brain barrier, activation of microglia, astrocyte proliferation, and the generation of proinflammatory cytokines.

MAMPs like LPS and peptidoglycans are more likely to enter the bloodstream through a leaky gut, triggering an immune reaction in the host. Toll-like receptor 4 (TLR4) localization and subsequent TLR signaling can both be induced by LPS [55]. Bacterial LPS can bind to TLR4 through a lipid-binding CD14 [56]. Numerous protein kinases, including myeloid differentiation factor 88 (MyD88), can be activated by this process. A range of chemokines, cytokines, and other immune factors can be produced when MyD88 is activated. Recent studies have found that activation of the TLR4/MyD88 signaling pathway promotes cellular injury in cerebral ischemic stroke [57]. Here, we outline the mechanisms driven by the bacteria that induce IS (Figure 2).

### 4.3. Neuroactive Pathways

Several neuroactive substances with host and microbial origins also play a crucial signaling role in host-microbiota interactions at the intestinal interface.

Catecholamines (CChs) include epinephrine, norepinephrine, and dopamine. CChs act as chemical neurotransmitters in the central and peripheral nervous system regulating various physiological processes and functions, including cognitive performance, intestinal motility, and mood [58]. *Escherichia* species, including commensals found in human guts, are known to produce CChs like norepinephrine. Norepinephrine exerts neuroprotective effects in the brain by reducing the transcription of inflammatory genes and boosting the creation of brain-derived neurotrophic factor (BDNF) by microglia and astrocytes, which can further enhance neuronal survival [59]. The gut produces more than 50% of the dopamine in humans, and gut bacteria can control peripheral dopamine levels. Also discovered in the biomass of *Escherichia coli*, *Bacillus mycoides*, and *Staphylococcus aureus* was dopamine [60]. The generation of cytokines by activated T cells and the activity of effector immune cells are regulated by dopamine [61]. Previous crossover experimental studies have shown that long-term levodopa administration in chronic stroke patients significantly improves motor performance in chronic stroke patients [62]. However, a recent study by Ford et al. showed that co-careldopa does not improve walking after stroke [63]. Therefore, more research is required to fully comprehend CChs dynamics in the gut lumen and its effects on mucosal immunity.

GABA is the main inhibitory neurotransmitter in the CNS. It was found that the introduction of a GABA-producing *Bifidobacterium* strain was adequate to regulate GABA levels in the gut [64]. GABA-mTORC1 signaling facilitates gut IL-17 expression when GABA levels are elevated in the jejunum of mice [65]. The study found that GABA selectively stimulates mucin-1 expression in epithelial cells. Precisely, GABA exposure to epithelial cells reduced IL-1*β*-mediated inflammation, increased tight junctions, and transformed growth factor beta (TGF-*β*) expression, which had a protective impact against the breakdown of the intestinal barrier [66]. Within the ENS, GABA, particularly the GABA_A_ receptor system, plays a role in neuronal excitability. It has been discovered that GABA_A_ receptors, in particular, mediate the suppression of T cell responses, which indicates GABA's potential role as a natural immunomodulator of T lymphocytes [67]. In experimental studies, activation of GABA receptors has been shown to have a neuroprotective effect in animal models of stroke by reducing infarct size and improving functional outcomes [67]. However, clinical trials did not support using GABA receptor agonists to treat acute stroke patients [68]. Further research is necessary to understand glutamate and GABA's relevance and functional dynamics as mediators of host-microbe interaction.

The cellular sources of histamine that have been best defined are mast cells, basophils, and histaminergic neurons. Cytokines such as IL-1, IL-12, IL-18, TNF-*α*, and calcium ionophores affect histidine decarboxylase (HDC) activity [69]. It has been demonstrated that histamine from *Lactobacillus reuteri* suppresses human monocytoid cells' ability to produce TNF-*α* in response to TLRs through signaling through the histamine H2 receptor and downstream cAMP and PKA activities [70]. However, a recent study in aged mice following experimental stroke showed that stroke resulted in increased intestinal mast cell numbers and intestinal histamine receptor expression levels. In the peripheral circulation, these gut-centered changes were linked to increased histamine levels and other proinflammatory cytokines (such as IL-6, TNF-*α*, and IFN-*γ*) [71]. It is obvious that histamine mediates host-microbe crosstalk as a neuroimmune system; however, it is still unclear how host histamine may affect microbial activity.

It has been demonstrated that serotonin (5-HT) functions as a neuroendocrine signal of host-microbe crosstalk, modulating bacterial motility, biofilm formation, exopolysaccharide production, and inducing the expression of virulence genes in bacteria via a quorum sensing mechanism [72]. Exogenous serotonin treatment worsened pathogenic intestinal symptoms, increased the formation of biofilm on mouse guts, and raised the release of proinflammatory cytokines. Serotonin levels in the blood and colon were lower in GF mice, and the brain's serotonin turnover rate was higher [73]. Serotonin produced by the gut microbiota in mammals can operate locally in the GI tract or reach the bloodstream, but it cannot cross the BBB [74]. However, serotonin has been shown to promote BBB permeability, indirectly affecting brain function [75].

### 4.4. Hypothalamic–Pituitary–Adrenal Axis Pathways

One of the primary neuroendocrine systems in the human body is the hypothalamic–pituitary–adrenal (HPA) axis, which also functions as a critical nonneuronal communication link in the MGBA [76]. The HPA axis and its neurotransmitter counterpart cause several changes in the neuro-immune system that prepare the body for the “fight or flight” reaction to stress [77]. Interactions between the immunity-HPA axis have been linked to a number of inflammatory and stress-related illnesses. The HPA axis is finally activated when exposure to microbes and antigens outside the epithelial barrier triggers the mucosal immune response [78]. The HPA axis functions can be regulated by stress response by modulating the expression of BDNF, the 2A subtype of N-methyl-D-aspartic acid (NMDA) receptors, and the hippocampus. A key component of the stress response is the cortisol. The HPA axis controls the release of CChs, mineralocorticoids, or glucocorticoids in response to stress to modify the intestinal microenvironment [79]. The corticosterone-releasing factor (CRF) in the hypothalamus can be altered by gut microbiota. Serum cortisol levels have been linked to poststroke mortality and severity [80]. The higher stress response in GF mice was slightly alleviated by fecal microbiota transplantation (FMT) and was entirely reversed over time by *Bifidobacterium* treatment [81]. Corticosterone or cortisol levels were lower in preclinical and clinical investigations following probiotic or prebiotic administration [82]. As a result, the HPA axis, a crucial regulator of the stress response, can affect how the MGBA is regulated.

### 4.5. Role of Gut Microbial Metabolites

#### 4.5.1. Trimethylamine N-Oxide (TMAO)

Through the action of intestinal microbial TMA lyases, trimethylamine (TMA) is generated from dietary nutrients (choline, L-carnitine, and phosphatidylcholine) [83]. The host's hepatic flavin-containing monooxygenases (FMOs) convert TMA to trimethylamine N-oxide (TMAO) [84]. Risk factors for recurrent IS and cardiovascular events with elevated TMAO levels were strongly associated with the levels of proinflammatory monocytes (CD14++/CD16+) [85]. When TMAO stimulates platelets, there is a potential for thrombosis due to an increase in the release of Ca2+ from intracellular reserves and platelet-dependent hyperreactivity [86]. Another study showed that after a choline stimulation test, intestinal symbionts transplanted from donors with high or low circulating TMAO levels had different effects on mice with arterial damage in terms of platelet reactivity and thrombosis potential [87]. Zhu et al. [88] discovered by microbial transplantation that the gut microbe CutC (an enzyme source for the conversion of choline-to-TMA) boosted host TMAO synthesis, enlarged the infarct area in the brain and caused functional impairment. In addition, atherosclerosis is a risk factor for IS. Wang et al. [89] reported that dietary supplementation with choline or TMAO increased atherosclerosis in *Apoe*−/− mice and promoted macrophage cholesterol accumulation and foam cell formation. Studies using animal models have shown that inhibiting FMO3 activity decreases TMAO levels and inhibits atherosclerosis [90]. TMAO increases the inflammatory responses in the vascular wall, causes the generation of reactive oxygen species (ROS), and inhibits the reverse transport of cholesterol, which leads to atherosclerosis [91].

#### 4.5.2. Short-Chain Fatty Acids (SCFAs)

Through bacterial fermentation, resistant starch and dietary fibers are degraded into SCFAs, such as acetate, propionate, butyrate, and other related compounds (5 carbons or less) [92]. SCFAs exert their effects by inhibiting histone deacetylases (HDACs) to influence gene expression and the ligands of the subset of G-protein coupled receptors (GPCRs) in the host epigenome [93]. Peripheral blood monocytes exposed to SCFAs showed reduced proinflammatory TNF-*α* production, inhibited NF-*κ*B activation [94], and decreased leukocyte adherence to endothelial cells by altering vascular cell adhesion molecules [95]. It has been demonstrated that SCFA stimulation of EECs causes the release of hormones such as glucagon-like peptide 1 (GLP-1) and peptide YY (PYY) [96]. This activity may initiate a signaling cascade that affects brain circuits that regulate appetite and inhibit gastric motility through the systemic circulation or vagal afferents. A prime illustration of a specialized intestinal cell that can act as a sensor for neurochemical signals derived from the microbiota and the CNS is the modulation of GLP-1 signaling by EECs. It enables regulating GI secretory activity, systemic immunity, obesity, diabetes, and other stroke risk factors. PYY affects brain activity and appetite by mechanisms that cross the BBB. Fermentable polysaccharides were added to human diets to raise plasma levels of PYY and GLP-1 [97]. Significantly fewer SCFA-producing bacteria, such as *Anaerostipes*, *Butyriciocus*, *Faecalibacterium*, and *Lachnoclostridium*, were present in patients with IS [98]. The decrease in SCFA levels may be partly due to poor prognosis in aged mice after stroke. Aged mice received a mixture of probiotics that produce SCFAs, including *Lactobacillus fermentum*, *Bifidobacterium longum*, *Clostridium symbiosum*, *Faecalibacterium prausnitzii*, and inulin, which resulted in an increased production of SCFAs and attenuated stroke-related neurological deficits and inflammation [99]. Dysbiosis of the intestinal flora linked to atherosclerosis revealed a decrease in butyrate producers such as *Eubacterium* and *Roseburia* in the intestine [100]. Experimental investigations have demonstrated that intestinal injection of butyrate lowers inflammation and atherosclerosis [101].

#### 4.5.3. Bile Acids (BA)

The chemical diversification of bile acids (BA) is the result of a cooperative effort between the host (synthesis of primary bile acids) and the gut microbiota (synthesis of secondary bile acids) [102]. BAs have been demonstrated to modulate systemic lipid metabolism, glucose metabolism, and cholesterol as well as immune homeostasis through the interaction of these amphiphilic molecules with membrane and nuclear receptors, including farnesoid X receptors (FXRs) and Takeda G-protein-coupled receptor 5 (TGR5) [103]. The main bacterial species in the gut, such as *Lactobacillus* and *Bifidobacterium* taxa, express the enzyme bile salt hydrolase (BSH), which deconjugates BAs from glycine and taurine [104]. According to research, intestinal inflammation [105], metabolic disorders such as diabetes [106], and cardiovascular disease [107] are pathologically determined by gut microorganisms and abundant BSH genes. Metabolite analysis of young stroke patients showed that their serum glycochenodeoxycholic acid (GCDCA) concentration was significantly higher than that of healthy controls [108]. Furthermore, it has been demonstrated that elevated taurocholic acid (TCA) and GCDCA concentrations following liver injury activate the glucocorticoid receptor (GR), which disrupts the hypothalamic–pituitary–adrenal (HPA) axis [109].

As a protective BA in brain diseases, tauroursodeoxycholic acid (TUDCA) has been thoroughly investigated for its anti-inflammatory and antioxidant properties [110]. TUDCA induces anti-inflammatory markers by binding to TGR5 [111]. Moreover, Cheng and colleagues discovered that TUDCA might be linked to suppressing oxidative stress in the brain [112]. Oxidative stress is a pathophysiological process after stroke and is closely associated with neuroinflammation, excitotoxicity, and apoptosis [113]. Compared to the untreated group, IS rats receiving a mixture of hyodeoxycholic acid (HDCA) and cholic acid (CA) had smaller infarcts and considerably lower TNF-*α* and IL-1 concentrations [114]. It has been noted that specific BAs metabolized by cytotoxic and hydrophobic gut bacteria, such as deoxycholic acid (DCA) and lithocholic acid (LCA), can worsen brain damage [115]. Another study discovered that decreased BA excretion may be an independent risk factor for IS and that greater DCA and LCA concentrations in fecal samples were related to higher poststroke survival [116].

#### 4.5.4. Tryptophan

In the digestive tract, there are three main pathways for tryptophan metabolism: (1) the gut microbiota's direct conversion of tryptophan into several compounds, including aryl hydrocarbon receptor (AHR) ligands [117]; (2) the indoleamine 2,3-dioxygenase (IDO) 1-mediated kynurenine pathway in both immunological and epithelial cells [118]; and (3) the Trp hydroxylase 1- (TpH1-) mediated serotonin (5-HT) synthesis pathway in enterochromaffin cells [117]. Intestinal microorganisms can metabolize tryptophan to produce molecules. For instance, tryptophan is used by *Escherichia coli* (*E. coli*) to produce indoles, which have beneficial effects on the intestinal flora and can lessen the biofilm and virulence development of *E. coli* and other bacteria [119]. In the intestinal microbial environment, indole is a significant intercellular signal that interacts with the intestinal epithelium by enhancing tight-junction resistance and the expression of anti-inflammatory cytokines [120]. The activation of astrocytes and microglia is regulated by tryptophan metabolites, which also govern neuroinflammation via AHR signaling [121]. Patients with acute ischemic stroke (AIS) and carotid stenosis reported lower concentrations of tryptophan and 3-hydroxyanthranilic acid (3-HAA) and higher levels of circulating arachidonic acid (AA) and 3-hydroxykynurenine (3-HK) in their blood than those of controls [122]. The ratio of kynurenine to tryptophan and stroke severity were positively correlated in a study of IS patients [123]. More research is required to fully understand how microorganism-derived tryptophan metabolites are linked to inflammation in brain illness.

## 5. Gut Dysbiosis and IS Risk Factors

In addition to being closely linked to gastrointestinal disorders such as irritable bowel syndrome and ulcerative colitis, an imbalance in the gut microbiota is also linked to the occurrence and progression of aging, hypertension, diabetes, obesity, and atherosclerosis, all of which are risk factors for IS [124].

### 5.1. Gut Microbiota and Aging

The incidence of stroke increases with age, with persons over 65 accounting for 70–80% of all IS [125]. Scientists can now examine alterations in the gut microbiota of elderly individuals because of recent developments in next-generation sequencing (NGS) and metagenomic technologies [126]. IS is mostly an aging-related disease, and aged mice do worse than younger mice when they experience an experimental stroke [127].

Recent research using experimental animal models has revealed that older mice tend to be more susceptible to infection after IS, which is at least partially caused by decreased intestinal barrier integrity and intestinal inflammation, as well as reduced expression of mucin and tight-junction proteins, which facilitates bacterial translocation [71]. Aged mice had a worse prognosis after IS than young mice. Crapser and colleagues demonstrated that IS causes intestinal permeability and bacterial translocation in young and aged mice. Young mice are able to resolve these issues, whereas aged mice experience prolonged sepsis and worse functional recovery following IS [128]. The unavoidable biological aging process significantly increases the risk of stroke and is linked to significant alterations in the gut microbiota composition. Specifically, aging is associated with significant decreases in *Firmicutes* and *Bifidobacterium* and increases in *Bacteroidetes* and *Proteobacteria*, particularly *Gammaproteobacteria* [129]. Another study showed that elderly mice had a *Bacteroidetes*/*Firmicutes* ratio that was nine times higher than that of younger mice. Young mice that received FMT from an elderly donor had higher levels of systemic proinflammatory cytokines and higher mortality after middle cerebral artery occlusion (MCAO). In contrast, FMT from young to elderly mice increased survival, enhanced motor strength after recovery from proximal MCAO, and improved motor function and anxiety [130].

These findings indicate that aging affects poststroke functional outcomes and survival by increasing gut dysbiosis. To potentially enhance stroke outcomes and recovery, it may be possible to alter the age of the gut microbiota, especially in elderly individuals.

### 5.2. Hypertension

The development of IS by hypertensive diseases is influenced by endothelial dysfunction, increased shear stress, and stiffness of the large arteries that transport pulsatile flow to the cerebral microcirculation [131]. Experimental studies have validated the role of the intestinal microbiota in the emergence of hypertension. First, giving germ-free recipient mice the gut microbiomes of hypertension patients raises their blood pressure [132]. Furthermore, germ-free mice exhibit decreased renal and vascular immune cell infiltration after receiving angiotensin II injections. They are also resistant to hypertension and vascular dysfunction [133]. Yang et al. [134] discovered an imbalance in the hypertensive rat microbe populations, which decreased overall, while *Bacteroides* numbers were elevated. Human research suggests that intestinal mucosa shape, gut-derived metabolites, and microbial taxa are all linked to hypertension. Compared to normotensive people, hypertensive people exhibit a microbial shift with an increased abundance of pathogenic taxa and lower microbial richness and diversity [135].

It has recently been found that excess Na+ can even be detected in intestinal microbiota and immune cells, which can promote inflammation and hypertension; an excessive amount of salt causes organ damage to the kidneys, vasculature, and CNS [135]. Low-sodium dietetic control is advised in poststroke therapy to promote healing and lower the chance of stroke recurrence. A high-sodium diet in mice reduces the *Firmicutes* to *Bacteroidetes* ratio, fosters gut barrier dysfunction, and damages inflammatory reactions [135]. Inflammatory pathways are controlled by the gut microbiota, which also plays a role in the etiology of hypertension. By raising the number of proinflammatory Th17 cells in the spleen, high salt intake also decreases the population of *Lactobacillus murinus*. In treated rats, daily injection of *Lactobacillus murinus* reduces Th17 cells and lowers blood pressure [136].

Gut microbiota-producing metabolites such as TMAO and SCFAs are crucial in developing hypertension. In animal models of hypertension, SCFAs reduce blood pressure and gut dysbiosis and restore the balance between Th17 and Treg cells [137]. SCFAs may modulate the SCFA receptor G-protein coupled receptor 43 (Gpr43) to promote Th1 but restrict Th17 cell differentiation to control blood pressure [138]. An accurate study of individual species and their metabolic products is needed to further our understanding of the gut microbiota and blood pressure management after stroke since the impacts of various metabolites may cause the disease to proceed in different directions.

### 5.3. Diabetes Mellitus/Obesity

The risk, prognosis, and outcome of IS are often worse for diabetic individuals than for nondiabetic individuals. Many stroke patients also have hyperglycemia even if they have no history of diabetes [139]. Two large-scale metagenome analyses in China and Europe examined the gut microbiota of type 2 diabetes (T2D) patients and healthy individuals. These two studies showed an increase in *Clostridium hathewayi* and a decrease in *Roseburia* in patients with T2D [140, 141]. It has been proposed that diabetes may decrease SCFA production or uptake, particularly the anti-inflammatory butyrate [141]. A recent study uncovered a causal link between the insulin response and a genetically driven rise in butyrate synthesis in the host. Conversely, propionate production or absorption irregularities have been linked to a higher incidence of diabetes [142].

GLP-1 produced by EECs in the gut microbiota regulates satiety and hunger. It is possible to distinguish between people with and without diabetes using the transcriptome signature of EECs in obese subjects. Notably, obese diabetic individuals exhibited decreased plasma GLP-1 as well as proglucagon maturation and GLP-1 cell differentiation [143]. An excellent illustration of a specialized intestinal cell that can act as a sensor for neurochemical signals derived from the microbiota and the CNS is the modulation of GLP-1 signaling by EECs. This allows for the reconciliation of known risk factors for IS, such as gastrointestinal secretion function, obesity, and diabetes [124]. Obesity is a significant risk factor for T2D. Experiments with fecal microbiota transplantation have shown that the gut microbiota is crucial for insulin resistance, adipose tissue accumulation, and energy absorption [144]. Several studies have also revealed considerably lower *Firmicutes*/*Bacteroides* ratios in the intestines of obese people and rats [145].

### 5.4. Atherosclerosis

Atherosclerosis is intimately associated with arterial stiffness, which results from the loss of elastic fibers and the thickening of arteriole walls. The accumulation of cholesterol in the arterial wall causes macrophages to phagocytose lipoproteins, forming foam cells characteristic of atherosclerosis [146]. Compared to asymptomatic controls, patients with atherosclerotic stroke had a different gut microbial composition, specifically an increased abundance of opportunistic pathogens (e.g., *Enterobacter* and *Desulfovibrio*) and a decreased abundance of commensal or beneficial genera (e.g., *Prevotella* and *Faecalibacterium*) [27]. Karlsson and colleagues discovered an increased abundance of the genus *Collinsella* in stool samples from individuals with symptomatic atherosclerosis (cerebrovascular events), whereas *Eubacterium* and *Roseburia* were enriched in controls, implying the existence of dysbiosis in atherosclerosis [147]. Patients with vascular disease frequently have oral *Streptococcus mutans* in their atherosclerotic plaques [148]. Additionally, it has been discovered that the oral bacterium *Porphyromonas gingivalis* is linked to the onset of IS [149].

One of the metabolites produced by the gut microbiota, namely, TMAO, has undergone extensive research and has been positively linked to the development of early atherosclerosis [88]. The current study suggests that TMAO can exacerbate brain damage following IS through many pathophysiological mechanisms. It can also induce vascular inflammation and endothelial dysfunction, increasing atherosclerosis and thrombogenesis [150].

Few studies have focused on the connection between atherosclerosis, bacteria in plaque, and the gut microbiota in individuals with IS. There is still much to study about how the gut microbiota influences the pathophysiology of atherosclerosis.

## 6. Intervention and Management Strategies for Ischemic Stroke That Target the Gut Microbiota

**Figure 3:**
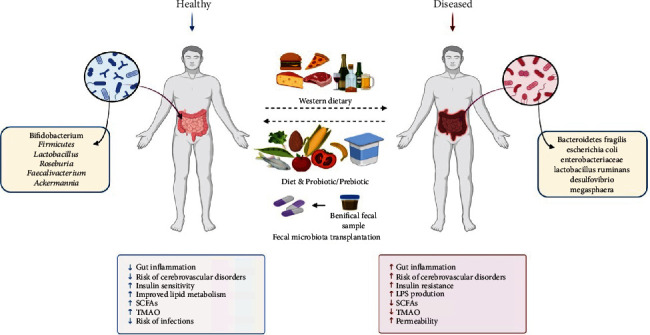
There are several ways to control IS by restoring the dysbiotic gut. A few strategies to treat gut dysbiosis include dietary modification by adding nutritious ingredients boosting the colonization of good bacteria in the gut using probiotics or prebiotics and FMT.

The findings of investigations on both humans and animals point to the possibility that gut dysbiosis may be a risk factor for the occurrence, severity, and prognosis of IS in patients. Emerging treatment approaches aim to reestablish a healthy gut flora to prevent and treat IS. Nutritional therapies that modify the gut microbiota to a healthy condition by diet, probiotics, prebiotics, and FMT through normal donors may aid in preventing the pathogenesis of IS (Figure 3).

### 6.1. Diet

It is evident that diet is one of the significant indicators of the intestinal microbiota and that changes in dietary habits can directly impact its composition, variety, and metabolic capacity through the availability of macro- and micronutrients in the intestine [151]. Animal-based protein increases the number of potentially harmful gut microbes in mice, such as *Escherichia*, *Ruminococcaceae*, and *Streptococcus*. In contrast, plant-based proteins increase the abundance of *Lactobacillus* and *Bifidobacterium* and decrease the number of pathogens, such as *Bacteroides fragilis* [152]. In addition, glycated pea protein ingestion increased microbial SCFA synthesis, which is known to have anti-inflammatory and intestinal barrier-protecting properties [153]. More precisely, there were noticeable differences in the relative abundance of specific bacteria related to different protein sources.

It is widely acknowledged that eating a high-fiber diet fosters bacterial diversity, increasing beneficial bacteria, including *Roseburia*, *Prevotella, Faecalibacterium*, and *Ackermannia*, while reducing possibly pathogenic bacteria (such as *Enterobacteriaceae*) [154]. In their study of 178 older persons, Claesson and colleagues discovered that those who consumed a high-fiber diet produced more butyrate and acetate from SCFAs than those fed a low-fiber diet [155]. Consequently, a high-fiber diet may stimulate the production of anti-inflammatory cytokines in the intestines and reduce brain damage following a stroke [156].

Caesar and colleagues compared the intestinal microorganisms in GF mice fed lard (saturated fatty acids) or fish oil (polyunsaturated fatty acids). They discovered that the fish oil consumption group had higher diversity and abundance of *Akkermansia muciniphila* and *Lactobacillus*. In contrast, the lard-fed group had a significant amount of *Bilophila wadsworthia* and showed evidence of Toll-like receptor 4 (TLR-4) activation [157]. This finding is consistent with earlier studies using saturated fats in clinical and experimental settings [158].

The gut microbiota releases small-molecule metabolites from food derivatives and microbial fermentation into the circulation, where they interact with the host and cause various illnesses, including IS. It is critical to comprehend how certain macronutrients, particularly dietary lipids, carbohydrates, and proteins, affect the gut flora.

### 6.2. Probiotics and Prebiotics

Live microorganisms known as probiotics, which are beneficial to the host's health, are primarily made up of *Bifidobacterium* and lactic acid-producing bacteria such as *Lactobacillus.* Bacteriocins, which are synthesized and secreted by probiotics, prevent bacterial invasion and block pathogens from adhering to epithelial cells [159]. Additionally, they exert a trophic impact on the intestinal mucosa and affect the secretion of cytokines from epithelial cells, which increases barrier integrity and mucus formation [160]. Furthermore, probiotics enhance GLP-1R expression in the brain and GLP-1 and 5-HT secretion in the intestine by maintaining the barrier's integrity [161]. Prestroke treatment of probiotics in mice inhibited TNF-*α* production, decreased neuronal damage to the hippocampus, and enhanced antioxidant enzyme activity [162]. Probiotics may have an effect by interacting with Toll-like receptors in intestinal epithelial cells to inhibit TNF-*α* and free radical production [163]. According to a meta-analysis, probiotic supplementation for stroke patients was linked to reduced serum TNF-*α*, IL-6, and IL-10 levels and positively affected poststroke recovery [164].

Prebiotics are a group of carbohydrates, such as resistant starch, oligosaccharides, especially fructose (such as inulin and fructooligosaccharides (FOS)), and galactose (such as galactooligosaccharides (GOS)), that are not digested by the host but can selectively alter the microbiome's activities and composition [165]. Rats treated with probiotics (B-GOS) exhibited improved cognitive performance, inhibition of microglial activation, and decreased expression of proinflammatory cytokines [166]. Similarly, the elderly population who consumed probiotics had significantly higher amounts of *Bacteroides* and *Bifidobacterium*, elevated lactate levels in their feces, a significant decrease in proinflammatory cytokines, and an increase in anti-inflammatory cytokines (IL-10 and IL-8) [167]. Studies have demonstrated that inulin boosts butyrate synthesis while promoting the production of *Bifidobacterium* and *Faecalibacterium* [168].

Therefore, it appears that probiotics and prebiotics are positive therapeutic alternatives for neurological illnesses, as suggested by these studies.

### 6.3. Fecal Microbiota Transplantation

Fecal microbiota transplantation (FMT) transfers intestinal microbiota from one healthy donor to another by oral intake of fecal matter in rodents or colonoscopy in humans. The FMT method has gained much attention for its significant percentage of success in the treatment of recurrent *Clostridium difficile* infection [169]. In a recent study, the effectiveness of FMT was compared using three different pretreatment strategies: antibiotics, bowel cleansing solution, and no pretreatment. The study indicated that antibiotic pretreatment increased the efficacy of FMT [170]. Recolonization by FMT in mice with MCAO with either healthy sham control gut microbiota or gut microbiota treated with antibiotics lessened brain injuries following an experimental stroke [20]. Germ-free mice developed larger infarcts after a stroke, but when their guts were colonized with normal gut microbiota, this condition began to improve [171].

Previous research has demonstrated that leaner donor FMT improves microbial diversity, insulin sensitivity, and butyric acid-producing bacteria in obese patients [172]. Diabetes is one of the major risk factors for IS. FMT of T2D-related gut microbiota in GF mice showed that dysbiosis of the gut flora increased brain damage aggregation and poststroke infarct size. It also caused intestinal barrier dysfunction in GF mice, including increased serum LPS and impaired tight-junction protein distribution [173].

FMT has the advantage of being coupled with other techniques for gut microbiota remodeling. However, FMT also has drawbacks, such as the fact that most studies use only animal models and that study designs vary widely, using various techniques, donors, and antibiotics. The FMT investigations did not identify any solid mechanisms underlying these advancements, so further research is needed to determine the mechanisms underlying this advantageous effect.

## 7. Conclusion and Future Perspectives

The importance of MGBA in a person's health status has been highlighted by numerous studies that found imbalances in the GI microflora composition to be related to particular abnormal physiological situations. The biological relationship between the gut microbiota, the CNS, and immune signaling suggests that systemic microbial signaling or microbial-derived metabolites may indirectly or directly impact immunological and neurological activity in IS. More research is necessary on the potential function and precise mechanism of MGBA in IS. It will be essential to enhance preclinical studies of novel therapeutics for the prevention and treatment of IS and to help understand the high risk of IS and its tendency for recurrence. Overall, it must be appreciated that the GI microflora could be viewed as a new organ and denoted the “second brain,” and that it plays a significant part in the pathogenesis of IS. Future research in neurotherapeutics will provide crucial information about the gut microbiota as a new dividing line between human health and disorders.

On the one hand, fundamental science research should employ randomization, blinding, and data analysis techniques similar to clinical trials to more accurately replicate the complexity of human trials. On the other hand, clinical studies should be sufficiently powered to evaluate efficacy in stroke subtypes, establish salvageable tissue and target engagement, and be mindful of the therapeutic window. Proteomics, metabolomics, and 16S microbiome sequencing should be used in future gut microbiome studies and IS to thoroughly understand the pathways, microbes, and metabolites involved and to clarify the effect of their interactions on IS. To conclude, the translatability of the results will increase if recent guidelines for bettering the quality of the design, collection, and analysis of microbiological datasets are followed, which is vital to advance the field and shift from association to causation.

## Data Availability

No data were used to support this study.
